# Dental Clinics in Public Institutions and in Public Schools

**Published:** 1923-01

**Authors:** Harvey J. Burkhart

**Affiliations:** Rochester, N. Y.


### DENTAL CLINICS IN PUBLIC INSTITUTIONS AND IN PUBLIC SCHOOLS

BY HARVEY J. BURKHART, D. D. S., ROCHESTER, N. Y.

*Mr. Chairman and Gentlemen:*

I feel very deeply the honor which has been done me during the sessions of the Alumni Association. I assure you it is a very great pleasure and privilege to come here and talk to the old boys of this institution, and while I have not attended very many meetings of the Alumni Association, and have not been in Baltimore very many times since graduation, it has not been due to a lack of interest in the school or in the men that I have been associated with who have come out of this college. I regard the friendships which I made while in college and since among the boys that have come out of here as some of the very warmest and very dearest of my professional life. There are just two things I want to talk about this morning. In my official position I am receiving letters all the time from men throughout the country, and of late from men in the South, asking my opinion with reference to the organization of dental clinics, especially those connected with public institutions and the public schools. There is no definite formula that I know anything about for the exact running of clinics of that nature, but it is my opinion that if you have not some one like Mr. Forsyth or Mr. Eastman, who will endow an institution and permit you to do general children's dentistry, then the only other place where this work can be done effectively is in connection with public schools.

On account of the increased cost of maintaining the public school systems, due to the increased cost of building, and especially the better pay teachers have been receiving the last three or four years, it is difficult to get boards of education to contribute very large sums for any innovation in the public school system. They are very reluctant to try anything new; first, on account of the economic question involved, and, secondly, because of a lack of understanding on the part of the public of the value of dental service. I think we have been remiss for a long while in not using such influence as we might have with our local school authorities in trying to educate them to a proper appreciation of the value of dental service. We have been more backward than those connected with other activities that usually work themselves into school systems.

One of the things that Mr. Eastman specified in establishing the Rochester Dental Dispensary was that the city should appropriate a certain sum of money for the doing of prophylactic work in the public schools. That was readily acceded to by the municipal authorities, and the first five years we receive $20,000, and the coming five years we shall receive $25,000. This work is all done under the direction of the dispensary. It does not involve any connection with the city so far as the running of the dispensary activities is concerned. The city authorities will never get a hand on the Rochester Dental Dispensary; that was safeguarded by Mr. Eastman and very wisely too.

We have squads of dental hygienists that are doing that work. When the work was started we sent squads of internes—recent graduates from the Dispensary. Those of you who have been thrown into close contact with the recent dental graduate can imagine something of the trials and difficulties we had in properly supervising the work. The slackness and slovenliness of some of the recent graduates does not speak very well for the appreciation of prophylactic work on their part. For the first two years we were obliged to use dental graduates. Then we organized a school for dental hygienists, and since that time we have had a sufficient number ahead to carry on the work without the use of the recent dental graduate except for the matter of supervision. Those who had rendered good service in the Dispensary for a year or two were usually chosen to take charge of squads of dental hygienists in the public schools, and since we have eliminated the dental graduate from work in the public schools, except for supervision, we have very much less trouble with the principals and teachers and the work has been more satisfactorily done.

The last year we have gone a step farther and we are now having the immediate supervising work done by the graduate dental hygienist instead of graduate dentists, and I can say to you, without disparaging the dental graduate in any way, we have had very much better and very much more loyal service from the young women who have had charge of the schools than we have ever had before. And while it may be thought that no supervision is given by licensed dentists, I want to assure you that there is. They are supervised by myself and graduate dentists who make daily visits to the schools where work is being done. After prophylaxis has been done in the various schools we go in and check up the work, so that we have a very good follow-up system and it is an easy matter to find out whether the work is being neglected or is being improperly done. My suggestion to any of you is that if you are not able to establish your dental clinics for the doing of general dental operations, which includes prophylaxis, you bend your efforts for the purpose of establishing clinics for the prophylactic work alone. I feel very certain if that were done in any community it would soon become so popular that you could supplement the work by the other branches of dental work which we all know is very necessary.

Without any reflection upon the men who live down South and their lack of activity in the obtaining of dental legislation for the promotion of dental clinics, and especially for the purpose of securing dental enactments which will permit the employment of dental hygienists not only in the schools but in private offices, I want to say that you are very much behind the times. The northern States for the last three or four years have been taking up the work with enthusiasm. We have a movement started that I think within the next two or three years will make operative the dental hygienist law in practically every northern State. I fancy from my conversation with some of you, and also from correspondence with others, that one primary reason for not being friendly to this proposed legislation is very largely due to the fact that it is the general thought of the dentist that the hygienist is going to interfere to a considerable extent with regular dental practice and, if I may speak more plainly, to take away some of the income of the dentist. The history of the States in which we have had the dental hygienist law is just the contrary. I have yet to hear of a single case where any dental hygienist has been charged with violation of the dental law, or of doing work in a field for which she is not educated. I want to say, further, in my opinion, that if the dental hygienist becomes an illegal practitioner of dentistry it is going to be due to the fault of the man by whom she is employed, or to his avariciousness in wanting her to make money for him. So I want to emphasize and drive home just as hard as I can this one thought, and that is that you should not be afraid of the dental hygienist coming into your State to practice, because she is not going to do you any harm and I know she is going to be a real value to you and to your patients, more than many of you now appreciate.

I practiced dentistry actively for over twenty-five years and have had the reputation of being a fairly decent operator, and gave my patients at least ordinary attention, but I am perfectly free to tell you that my patients did not receive the attention they should have received, and the prophylactic work was not done by me in the manner that it should have been and would have been had I had a dental hygienist in the office. That is due to two or three reasons. First of all, our patients have not been educated to pay a proper fee for the doing of prophylactic work in a proper way, and the other, we have been too busy. The extra prophylactic work we expected to do we put off from time to time thinking that we would find a more propitious time. I think those reasons are perfectly reasonable, and I think they will appeal to you. I know that my patients did not receive the attention, so far as the proper care of the mouth is concerned, that they were entitled to, and that I should have given them.

The dental hygienist can pay her way and she can make money for you if you want her to, and at the same time can render you very good service. I think no one should fear the dental hygienist on account of any loss of business. She is very valuable to you in many ways, such as keeping your records, looking after your office, and she will do more in the spreading of the gospel of oral hygiene than you will have time to, or will do. It is in line with the natural instincts and trend of a woman's work to be cleanly, and your offices will be better kept.

I should feel very well repaid if anything I may be able to say to you would induce you men to get busy and try to see that the legislation is obtained in the States where it is not now in existence, and to carry on this work, as I feel sure it would not be carried on and can not be without the assistance of the dental hygienist.

I want to thank you for your attention, and say that if there is any service I can render any of you in the way of furnishing you with suggestions for a dental hygienist law; if I can send you blanks, statistics, or a detailed statement of the work which we are doing, to guide you for use in arguments with your school authorities and your boards of aldermen, or with your Legislature, I shall be very glad to place anything we have at your disposal.—*Dental Cosmos.*

